# A qualitative study of abortion decision-making trajectories among pregnant women at their first antenatal care visit in Kampala, Uganda

**DOI:** 10.7189/jogh.15.04125

**Published:** 2025-04-11

**Authors:** Blake Erhardt-Ohren, Alison M El Ayadi, Hadija Nalubwama, Carol S Camlin, Dilys Walker, Josaphat Byamugisha, Alexander C Tsai, Umar Senoga, Paul J Krezanoski, Cynthia C Harper, Alison B Comfort

**Affiliations:** 1 School of Public Health, University of California Berkeley, Berkeley, California, USA; 2 Department of Obstetrics, Gynecology, & Reproductive Sciences, University of California San Francisco, San Francisco, California, USA; 3 School of Medicine, College of Health Sciences, Makerere University, Kampala, Uganda; 4 Center for Global Health and Mongan Institute, Massachusetts General Hospital, Boston, Massachusetts, USA; 5 Harvard Medical School, Boston, Massachusetts, USA; 6 Department of Epidemiology, Harvard T.H. Chan School of Public Health, Boston, Massachusetts, USA; 7 Zuckerberg San Francisco General Hospital, University of California San Francisco, San Francisco, California, USA

## Abstract

**Background:**

In Uganda, only about half of women who want to avoid pregnancy are using modern contraceptives, leading to high numbers of unintended pregnancies and elevated maternal and neonatal morbidity and mortality. In this study, we aimed to learn more about women's abortion decision-making before continuing to carry a pregnancy.

**Methods:**

We utilised a qualitative study design and interviewed 31 purposively selected single and partnered pregnant women aged ≥18 years at their first antenatal care visit at Kawempe National Referral Hospital in Kampala, Uganda. We conducted the interviews in Luganda or English, transcribed them, and then translated them into English, as needed, for analysis. We analysed the data using thematic analysis. Deductive codes were based on social networks, social support, and health behaviour theories, and inductive codes were derived from interview transcripts.

**Results:**

Almost half of the study participants (n = 13) considered an induced abortion before deciding to continue carrying their pregnancy. The most commonly stated reasons they considered abortion included anticipated interruptions to work and education, exhaustion related to child-rearing, and lack of social support. Other participants (n = 9) reported not considering abortion due to anticipated social support for their pregnancy, concerns about abortion-related morbidity and mortality, late confirmation of pregnancy, and religious beliefs. No participants discussed Uganda's restrictive abortion policies as a reason not to consider abortion.

**Conclusions:**

Our results point to opportunities for continued reproductive health education and improved access to reproductive health services to allow pregnant women to meet their reproductive needs, seek out family planning, antenatal care, and safe abortion services when desired, and create support networks for pregnant women.

In 2022, a nationally representative survey in Uganda found that 42% of married women of reproductive age wanted to have another child later, 33% were undecided, 7% wanted another child but were undecided when, and only 15% wanted to have another child soon [1]. Yet only 38% of these married women were using a modern contraceptive, suggesting that 22% of married women of reproductive age had an unmet need for family planning. Despite abortion only being legal ‘for the preservation of the mother’s life, if the performance of the operation is reasonable, having regard to the patient’s state at the time, and to all the circumstances of the case’ [2], abortion still occurs in Uganda. Among the estimated 2.4 million pregnancies in Uganda annually in 2015–19, approximately 58% were unintended, and of these, an estimated 30% were terminated [3], meaning that nearly one million unintended pregnancies are carried to term.

The potential adverse consequences of carrying a mistimed and/or unwanted pregnancy to term and not being able to access safe abortion services are well-documented. Unsafe abortion is one of the leading causes of maternal morbidity and mortality in Uganda [4], accounting for approximately 18% of deaths among women aged 15–49 years [5]. The maternal mortality ratio is estimated at 189 deaths per 100 000 live births [6]. Among women who experience medical complications during pregnancy and delivery, an estimated one-third receive the needed care for them or their infant [7]. Moreover, unintended pregnancy is associated with an increased risk of sexual violence from the pregnant woman's partner [8–11] and pregnancy complications [12,13]. There may also be mental health repercussions for pregnant women with unintended pregnancies who cannot access abortion services: several studies have found that women who are denied an abortion are most at risk for poor mental health and socioeconomic outcomes [14–16] and significantly more likely to experience depression during pregnancy [11] and postpartum depression than women with wanted pregnancies [11,17]. Infants that are the result of unwanted pregnancies are significantly more likely to be born preterm [11] with low birth weight [11,12] and less likely to be breastfed [11,12], all of which are associated with worse long-term development and health outcomes for the child [18–22].

There are complex, multifactorial influences contributing to decisions around abortion. Two recent studies in high-, middle-, and low-income countries reported that fears of financial consequences and/or interrupted educational endeavours are commonly reported primary reasons women pursue abortion [23,24]. Lack of community and partner support for pregnant women may also contribute to abortion considerations; several studies have reported that there is a belief that community support is necessary for healthy pregnancies [25] and child-rearing [26], and that single parents raising children may face additional burdens [27] in sub-Saharan Africa. Additional research has identified the stress and conflict that arises between sexual partners with unintended pregnancies in this context [28,29].

Only a few studies in low- and middle-income countries have investigated pathways to abortion decision-making and procurement [30–33]. There are no studies in low- and middle-income countries, to our knowledge, that have explored women's decision-making around abortion before deciding to continue to carry a pregnancy. Such information is crucial for antenatal care practitioners to better provide their patients with the knowledge and tools necessary for them to make informed decisions about their reproductive health. Given the restrictive abortion landscape in Uganda, the high number of unintended pregnancies and high rates of maternal mortality and morbidity, and the importance of reproductive autonomy, we conducted in-depth interviews with pregnant women seeking antenatal care to learn more about their abortion decision-making trajectories in this setting with restrictive abortion laws.

## METHODS

### Study setting

We conducted this study at Kawempe National Referral Hospital in Kampala, Uganda, with 31 pregnant women seeking their first antenatal care (ANC) service. Kawempe National Referral Hospital is the public referral hospital for the Kampala metropolitan area and has an outpatient clinic for ANC.

### Participants and recruitment

We recruited study participants from 25 August 2020 through 26 October 2020, once in-person ANC visits resumed during the COVID-19 pandemic in Uganda. Pregnant women were eligible if they were aged ≥18 years, the age of majority, and visiting Kawempe National Referral Hospital for their first ANC visit, regardless of gravidity, parity, or gestational age. We did not include girls aged <18 years old due to parental consent requirements, which were difficult to collect during the pandemic and because of the sensitive nature of the research topic.

We purposively recruited two sub-groups – pregnant women who identified as being in a partnership (either married or cohabitating) and pregnant women who identified as single (never married, divorced, separated, or widowed) – so we could make comparisons within and across groups. Recruitment of partnered women was restricted to those who were attending ANC with their partner in person due to COVID-19 research guidelines in Uganda. Though we recruited women who identified as single at the time of their visit, the sexual partner involved in the pregnancy was often still part of these women's lives, which is demonstrated in some of the study results. This study was nestled within a parent study examining the role of social support for ANC-seeking and decision-making around when to commence ANC, and recruitment ended when thematic saturation was achieved after 31 interviews for the parent study.

### Data collection

We collected sociodemographic characteristics and, if applicable, prior pregnancies, self-reported gestational age, and quality of relationship with their partner, followed by a semi-structured, in-depth interview. Though we did not collect gender identity data, we refer to study participants in our sample as women, given that this is culturally the local understanding of how pregnant individuals would identify given the socio-cultural context in Uganda. The interview guide included questions that explored how the participants confirmed their pregnancy, with whom they disclosed their pregnancy and whether they delayed sharing that information; who the participants involved in decision-making about antenatal care-seeking and how those individuals (*e.g.* partner, family member, friend) facilitated or impeded access to pregnancy-related services through provision of social support; and whether the participants considered terminating their pregnancy, their reasons for deciding not to terminate the pregnancy, and who they relied on or avoided during this decision-making. Other areas of focus included factors that contributed to delays in ANC-seeking and women's autonomy with care-seeking decisions (Material S1 in the **Online Supplementary Document****)**.

Our research team recruited pregnant women from the ANC clinic waiting area. If they met the study eligibility criteria, the research team invited them to participate in an interview after finishing their ANC visit. Following the visit, the team obtained participants’ written consent for study participation (if literate) or verbal consent in the presence of a literate, impartial witness (if they were not literate). One trained interviewer (HN) conducted each in-depth interview in either Luganda, the local language, or English (depending on participant preference). All interviews were conducted in a private room. They were recorded, and the interviewer took clarifying notes. Interviews lasted between 70–90 minutes. Study participants received UGX 20 000 (approximately USD 5) for the time required for their participation in the interview, consistent with other research studies in this area. After the interviews, the research team transcribed and then translated the interviews into English for analysis. The research team also reviewed the study participants’ ANC records to confirm their gestational age at this first ANC visit.

Participants in the study were interviewed by a female interviewer (HN) who was the research coordinator. HN has 12 years of experience in conducting qualitative research and has a Master of Public Health degree.HN built rapport with study participants before conducting the interviews and while obtaining informed consent. She introduced herself to all study participants and detailed the reason why the study was being conducted.

### Data analysis

After the first 10 interviews were completed, the research team transcribed and translated the interviews, reviewed the data, and adjusted the interview guides to ensure that relevant themes, concepts, and ideas were fully explored. At this point, we also adapted recruitment to ensure greater heterogeneity in parity. After we completed, transcribed, and translated 21 additional interviews, we imported all of the data into ATLAS.ti, version 8 (Lumivero, Denver, Colorado, USA) for complete analysis. For the parent study, we employed a two-stage coding process utilising thematic analysis [34,35]. First, one researcher (AC) developed deductive codes derived from key constructs from the social networks and health behaviour framework of Berkman et al. [36] and the domains of the social support framework of Cohen and Wills [37]. The same coder then reviewed the transcripts and added inductive codes as themes emerged through line-by-line coding. This coder then created a codebook with definitions for both inductive and deductive codes. The codebook included codes specific to abortion decision-making which this current analysis used, such as ‘pregnancy termination,’ ‘pregnancy intentions,’ ‘decisions during pregnancy,’ and ‘feelings about pregnancy.’ AC, HN, and TR met to review the codebook. Thereafter, HN and TR independently coded the same four transcripts to ensure consistency. After reviewing discrepancies, HN and TR coded all remaining transcripts. and consistently met together with AC throughout the coding process to discuss coding questions and resolve any uncertainties around code definitions. For the purposes of this analysis, BEO used additional focused coding applied to the specific sections of the transcripts describing abortion-related considerations and decision-making. These focused codes were developed using inductive codes derived from the data.

### Ethical review

University of California at San Francisco Human Research Protection Program (number 254238) and the School of Medicine Research and Ethics Committee at Makerere University College of Health Sciences (2019-159) provided ethics approval, for both interviews and interview participants’ medical record reviews. Consistent with national guidelines, we also obtained clearance to conduct the study from the Uganda National Council for Science and Technology (SS 5163).

## RESULTS

Sixteen single pregnant women and 15 partnered pregnant women participated in the study. Most participants (n = 25) were aged <25 years. There were more young women aged 18–19 years (n = 8) among single participants than among partnered participants (n = 0). All participants reported at least some education, with most (n = 24) having received at least some secondary education. The participants’ religious identities were Anglican (n = 6), Catholic (n = 10), Muslim (n = 8), and Pentecostal or Born Again (n = 7). The majority of study participants were nulliparous (n = 20), and only two participants had two or more living children. Data from the study participants’ ANC records showed that six participants were in their first trimester of pregnancy, 15 in their second trimester, and 10 in their third trimester. Interestingly, women who considered abortion were approximately 23 weeks pregnant at their ANC visit and women who did not consider abortion were about 19 weeks pregnant (Table S1 in the **Online Supplementary Document**).

During interviews, 22 participants spoke about abortion. One single woman and two partnered women reported previous abortions. Almost half (n = 13) of the study participants said they had considered abortion before deciding to continue carrying their current pregnancy and attending their initial ANC visit. Nine women reported not considering abortion and provided reasons why they did not consider abortion. Participants who considered abortion and participants who did not consider abortion presented similar reasons for deciding to continue carrying their current pregnancy.

### Reasons for considering abortion

Many reasons for contemplating abortion were presented by 13 study participants who initially considered abortion before deciding to carry the pregnancy to term, spanning the domains of the socioecological model [38]. The reasons included interruptions to work and education, exhaustion related to child-rearing, not ‘liking’ the pregnancy, disagreements about child-rearing with their partner, and not having enough social support.

### Interruptions to work and education

The most common reasons for considering abortion, mentioned by about half of the women considering abortion, were related to work and education. When referring to work as a barrier to childbearing, women discussed their lack or their partner’s lack of employment or the difficulty in continuing to work while pregnant and how these challenges with employment would affect their own or the couple’s ability to provide financially for the child. For example, one participant, who was working as a bank agent at the time of the interview, stated:

I told [my partner about the pregnancy] because the pregnancy caught me off guard [when we were using] family planning… I thought I would give birth like two to three years to come but it happened [during the COVID-19 pandemic] and he wasn’t working. So I thought it was going to be hard on me because I almost failed to work after my first child. *– Partnered woman, 24 years, 28 weeks pregnant.*

Similarly, education was a concern raised by several participants due to their or their partner’s enrollment in school. One participant reflected on her first thoughts after learning of the pregnancy:

I was scared. I said to myself, ‘I am still in school...My partner is also still in school. *– Partnered woman, 20 years, 16 weeks pregnant.*

### Exhaustion related to child-raising

Several participants who considered abortion discussed their personal tiredness and feeling that they already had too much work taking care of another child. One of the participants, who had six living children, spoke about her conversations with family members about the pregnancy and stated that:

[Abortion] was just a thought on my heart, but one time I told my elder daughter that I feel so exhausted about this whole situation, and she told me, ‘Mother why? Mother be strong.’ When she said that to me, I got stronger. *– Partnered woman, 38 years, 12 weeks pregnant.*

### Other reasons

Many participants gave other reasons for considering abortion. They stated that they didn’t like the pregnancy, their partner did not believe that it was his child, their partner wanted to wait until they owned a home, and they did not have enough social support. Finally, a few stated that they did consider termination, but they did not elaborate on the factors influencing their decision-making.

### Reasons not to consider abortion or to decide to continue carrying the pregnancy after considering abortion

Pregnant women who did not consider abortion and those who decided not to pursue abortion shared concerns about injury or death from abortion, anticipated social support for the infant, religious reasons, and late confirmation of pregnancy. We found no differences in the reasons for carrying a pregnancy to term after considering an abortion between partnered and single pregnant women.

### Concerns about injury or death from abortion

Most participants reported not considering or pursuing abortion due to concerns about injury or death from the procedure. Several participants reported being warned by others, especially their mothers or partners, about potential adverse health consequences from abortion. One participant described her mother’s response:

I told her, mother, I am going to terminate the pregnancy. But she replied, if you abort it, it’s only you to get hurt not [your partner]. You will either die in the process or stay in a coma. *– Single woman, 18 years, 28 weeks pregnant.*

A few study participants reported either directly hearing stories or witnessing adverse events among other women following an abortion as a reason not to follow through with their own abortion. These women expressed concerns that they might experience the same outcomes as those whose stories they had heard or witnessed and were therefore hesitant to seek out abortion services. A few participants spoke about their own previous negative experiences with abortion, with one participant stating:

I would decide on [keeping the pregnancy or having an abortion] because it is related to my health; I might get complications [with an abortion]. I once had an abortion, and I didn’t like the condition I was in. That’s why I didn’t want to do it again. *– Partnered woman, 23 years, 12 weeks pregnant.*

These participants feared having the same injurious outcomes from their previous abortion or experiencing something worse.

### Support for the infant

Pregnant women who did not consider abortion, and more than half of the participants considering an abortion, discussed support they anticipated receiving from either a partner, family member, or the community to care for their infant. Several women initially felt they did not have the resources to care for an infant on their own and needed assurances from others to feel confident in the decision to carry a pregnancy to term. Single participants most often discussed the need for informal or formal marriage commitments from their sexual partner, whereas partnered women spoke about financial needs. One participant recalled a conversation they had with their sister when planning to terminate their pregnancy:

[My sister] supported [the decision to terminate]. She said ‘Giving birth is not easy. You are still in school. How will you attend lectures? And remember, men can leave anytime; you can wait until [your partner] does the traditional marriage.’ But [my partner and I] sat together, and he agreed to officially visit my parents. *– Partnered woman, 20 years, 16 weeks pregnant.*

This was a relatively common experience among participants contemplating abortion and participants who did not consider abortion. Several other participants, both partnered and single, also discussed the need for an informal commitment from the man involved in the pregnancy in order to feel comfortable bringing the pregnancy to term. These commitments would include emotional and financial support, as well as responsibility for the pregnant woman and the baby's physical health. When participants did not feel that they had support from the men involved in their pregnancy, they most often turned to their mothers for support, including financial, emotional, and instrumental support.

Several participants spoke of the negative responses and consequences that their partners, mothers, and other individuals in their support system spoke of when abortion was discussed. Negative responses ranged from the reporting of scolding and admonishments to verbal assault, and the pregnant women feared the consequences of being kicked out of houses, disowned, or exiled by their community. For example, one participant’s mother stated that:

‘...said that she will disown me if I aborted [the pregnancy]’ *– Single woman, 20 years, 16 weeks pregnant. *

Some participants spoke more generally about their concerns regarding raising a child without social support. However, once participants secured the support they felt that they needed to raise the child that would result from their pregnancy, they made the decision to bring the pregnancy to term.

### Religious influences on decision-making

Several participants discussed how their own or their partners’ religious beliefs conflicted with considering or procuring an abortion. A few participants believed that their pregnancy was the result of God’s will and therefore did not feel that they could make the decision to terminate their pregnancy. Similarly, another few participants were concerned that God might not allow them to become pregnant again if they terminated their current pregnancy. One participant summarised this belief by stating:

Since I got this pregnancy, I realised that this is what God has wanted for me, so there is no way I was going to abort. I feared this could be the only child I could have. *– Single woman, 23 years, 20 weeks pregnant.*

Only two women who shared that they initially considered terminating their pregnancy specifically discussed abortion as unacceptable in their religion. One participant recalled a discussion with her partner and shared:

You know, [my partner] is Muslim, so he read me a few scriptures and told me all the sins I will have committed. He told me that if the foetus hasn’t formed then it would be okay, but this one has formed, it’s a human being. *– Partnered woman, 24 years, 28 weeks pregnant.*

### Late confirmation of pregnancy

The other main reason that several participants did not consider or chose not to pursue an abortion was due to confirmation of their pregnancy during their second trimester. Delays in confirming their pregnancy were related to wanting to wait until they were confident that they were pregnant following missed menstruation. Many of the participants said they tested at home using home pregnancy tests but did not confirm their pregnancy with a medical provider until their first ANC visit through clinic-based testing. Of note, women who considered abortion were approximately 23 weeks pregnant at their ANC visit and women who did not consider abortion were about nineteen weeks pregnant, on average. One participant reported:

I thought about [abortion], but [the foetus] was way too grown to do it. I tried [inducing an abortion], but [the abortion] failed. *– Partnered woman, 24 years, 28 weeks pregnant.*

A few other participants shared that they would have wanted to know they were pregnant earlier so that they could have had more time to make the decision about whether to carry a pregnancy to term.

### Attempted procurement of abortion services

While most study participants ultimately decided to carry their pregnancy to term without seeking abortions services, a few participants spoke about their attempts to procure abortion services. None were successful in attaining an abortion. In a couple of cases, providers told the participants that they were too far along in gestation to receive an abortion. One of these women relayed their story:

[My partner] one time took me to a health provider and laid me in the bed, but I had no clue what they were up to. So, the health worker examined the pregnancy and told him, ‘Sir, you must raise your child because it has already formed. I can’t abort it.’ When we went back home, he beat me. He slapped me and I bled from the nose. *– Single woman, 19 years, 12 weeks pregnant.*

## DISCUSSION

This study fills an important gap in research on abortion considerations among pregnant women in Uganda who ultimately decided to continue their pregnancies, contributing to the literature around reproductive empowerment in this population. We found that although only three out of 31 study participants had sought abortion services for prior pregnancies, almost half considered abortion for their current pregnancy, and three attempted to procure abortion services for the current pregnancy before ultimately deciding to attend a first ANC visit. Pregnant participants considered abortion due to anticipated interruptions to work and education, exhaustion related to child-rearing, and lack of social support. Interestingly, and perhaps due to social stigma, no one discussed the potential adverse health consequences of carrying the pregnancy to term for the pregnant woman or the infant despite the persistently high rates of maternal and neonatal mortality and morbidity in Uganda [4–6]. Both pregnant women who considered abortion and those who did not mentioned concerns about abortion-related morbidity and mortality anticipated social support, religious beliefs, and gestational age at the time of pregnancy confirmation. Our study results point to the need for higher quality reproductive health education, increased social support for pregnant women, and improved access to high-quality family planning services, including contraception, early pregnancy confirmation, and safe abortion. This research points to the need to provide pregnant women with the knowledge and tools to make and act upon informed decisions about their health and well-being.

Overall, our findings show how common it is for pregnant women to consider abortion in a context like Uganda with high fertility, high rates of unintended pregnancy, high maternal morbidity and mortality, and where abortion is legally restricted [4–6,39]. These findings suggest the need for multi-pronged strategies to allow women and their communities to prevent unintended pregnancies, adequately space intended pregnancies, and seek out family planning and safe abortion services when desired. With 22% of women of reproductive age having an unmet need for family planning [1], there is a critical need to tackle the barriers preventing women from using their desired contraceptive method, with a focus on factors related to knowledge, attitudes, and access, as well as social norms and social support for family planning methods. There is also an opportunity to dispel myths around the danger associated with abortion; several studies have shown how effective and safe self-managed first-trimester medication abortion can be in restrictive policy environments, especially with accompaniment support for harm reduction [40–44]. These educational and service-level interventions can help pregnant women to feel empowered to exercise their reproductive rights and make decisions that are right for them at the time of their pregnancy.

In addition, our findings highlight the importance of social networks in providing support to pregnant women. Given pregnant women's concerns around the consequences of pregnancy for their education and/or work commitments and its influence on their finances, social support from their close social relations can be critical in their decision-making of whether to have another child. Greater social support for pregnant women could also alleviate concerns around child-rearing if that is their preference, including the physical, mental, and emotional consequences for the parent and household. There is also a role for community leaders, health care workers, and community health volunteers both in ensuring access to comprehensive reproductive health services while also mitigating social isolation during pregnancy.

Our findings are consistent with prior research, reviewed below, that has investigated factors contributing to abortion decision-making. Pregnant women in our study most commonly considered abortion due to the anticipated effect their pregnancy would have on their or their partner’s work and/or education. Participants often spoke about their or their partner's lack of employment or the difficulty a pregnancy would pose to continuing their education. Other studies confirm the importance of being able to keep working and/or complete education before having a child. A study in Ghana found that the most often reported reason for abortion among participants (36%) was the desire not to disrupt their education or employment [24]. A multi-country study looking at nationally representative data from 13 high, middle, and low-income countries found that, in six countries, socioeconomic concerns were the most common reason for obtaining an abortion, cited by 27–40% of respondents in each country [23].

Participants reported considering an abortion due to exhaustion related to child-raising and concerns about not having enough social support. Both single and partnered pregnant women discussed the need for assured commitments from their partners and family to help with raising a child (or another child) before deciding to carry the pregnancy. This finding aligns with previous research that demonstrates the multiple adversities faced by single parents raising children [27] and the importance of familial and community tangible and emotional support for healthy pregnancies [25] and child-rearing in sub-Saharan Africa [26]. Interestingly, the study participants did not discuss other social influences, such as family size or gender composition of existing children, which are important contributors to fertility preferences in Uganda and other high-fertility settings [45].

Participants who had considered terminating their pregnancy described many reasons for deciding to carry their pregnancy to term, which included concerns about injury or death from abortion, religious convictions, and late confirmation of pregnancy. Participants discussing abortion-related morbidity and mortality spoke about their own previous experiences with obtaining abortions and stories they had heard about others trying to obtain an abortion. There is very little published research exploring fears of undergoing an abortion procedure. Instead, the limited literature focuses on patient fears of social harassment prior to and following safe abortion and post-abortion care [46–49]. A few participants spoke about their pregnancy being God’s will or God removing their ability to become pregnant in the future if they procured an abortion. Again, there is little research exploring religion and abortion in Uganda from the patient's perspective and almost all literature focuses on provider perceptions of religion and its influence on reproductive health service provision [50–52]. However, fatalism has been found to be influential for other perinatal health-related behaviours in this setting [53–56]. Confirmation of pregnancy in the second trimester also dissuaded participants from pursuing abortion. While abortion is restricted regardless of gestational age in Uganda [2], participants described wanting to have confirmed their pregnancy earlier so they could have more time to decide whether to seek out abortion services and/or arrange support structures with their partners and families. Notably, no participants cited the restrictive legal environment or inability to find providers or abortifacients as a reason to continue their pregnancy and not pursue an abortion. This is not surprising – abortion rates are similar in countries where abortion is highly restricted and where it is broadly permissible.

### Limitations and strengths

This exploratory study has certain limitations and strengths. We only interviewed pregnant women aged ≥18 years and receiving care in an urban setting, meaning that our findings do not reflect the experiences of younger pregnant women and their decision-making in pregnancy, which could be quite different [57–59] or the experiences of rural-dwelling pregnant women who likely have much more limited access to care [60–62]. While it is likely that the pregnant participants in this study continued on to carry their pregnancy to term without procuring abortion services, it is possible that they sought out abortion after this first ANC visit; therefore, we are unable to report on pregnancy outcomes within this cohort. While we sought to attain variation in study participants’ parity, this was the first pregnancy for most study participants. In terms of our study’s strengths, the participants are diverse in terms of age, education, religion, and gestational age represented. This is also the first study of abortion decision-making trajectories among pregnant women at their first antenatal care visit within the Ugandan context. The qualitative design provides rich insight into this topic and points to the need for more targeted research to truly understand how pregnant women make decisions about abortion and child-rearing.

## CONCLUSIONS

We revealed the extent to which pregnant women may consider pursuing abortion before deciding to carry a pregnancy to term. The themes that arose in this exploratory qualitative study demonstrate the myriad interacting factors at the individual, interpersonal, health systems, and community levels that influence abortion decision-making. Our findings imply a need for high-quality reproductive health education, including training on harm reduction and accompaniment for induced abortion, strategies to increase social support for pregnant women, and ways to remove barriers to contraceptive services and early confirmation of pregnancy. Ultimately, these solutions respond to a human rights issue – ensuring that pregnant women can make informed decisions about their reproductive health.

## Additional material


Online Supplementary Document
