# Supplementary material for: A qualitative study of abortion decision-making trajectories among pregnant women at their first antenatal care visit in Kampala, Uganda
Source: J Glob Health. 2025 Apr 11;15:04125. doi: 10.7189/jogh.15.04125 (PMC11984614; doi:10.7189/jogh.15.04125)
Supplement: Online Supplementary Document [file jogh-15-04125-s001.pdf]

Supplement toErhardt-Ohren B, El Ayadi AM, Nalubwama H, Camlin CS, Walker D, Byamugisha J, Tsai AC,Senoga U, Krezanoski PJ, Harper CC, Comfort AB. A qualitative study of abortion decision-making trajectoriesamong pregnant women at their first antenatal care visit in Kampala, Uganda. J Glob Health 2025;15:04125.

Material S1. Complete interview guide

1. First, I would like to know more about the expectations in your community for <u>how men and women should behave during pregnancy</u>.
    a. How do you think members of this community expect <u>women</u> to behave when they become pregnant.
        i. *Probe: What expectations are there related to communication about pregnancy? Who is she supposed to talk with or not talk with about her pregnancy?*
        ii. *Probe: What expectations are there related to decision-making around pregnancy? Who typically gets involved in decision-making about seeking antenatal care?*
    b. How do you feel about those expectations for women?
    c. Now tell me more about how you think members of this community expect <u>men</u> to behave when they find out their partner is pregnant.
        i. *Probe: How are men expected to support their partner?*
        ii. *Probe: How are men expected to be involved in pregnancy-related decisions? What decisions are they expected to make on their own? What decisions are they expected to make with their partner? Which ones should she be making on her own?*
        iii. *Probe: In what ways is the partner expected to provide support (probe about emotional support, information, financial).*
        iv. *Probe: Are there areas where men aren't expected to be involved? Which ones? What are the reasons they shouldn't be involved?*
    d. How do you feel about those expectations for men?
    e. Aside from your partner, who are the people that you are expected to involve in decision-making throughout the pregnancy (e.g. mother, partner, sister, mother-in-law, friends, others) What are the reasons you are expected to involve them? What are your feelings about these expectations?
        i. In what ways are these people expected to be involved? Probe specifically about: emotional support, financial support, information, other support.

2. Now, I would like to hear <u>how your pregnancy has been going</u>. What has this pregnancy been like for you?
    a. *Probe: What has been easy about this pregnancy? What has been difficult about this pregnancy?*

   b. *Probe: If pregnant before – how has this pregnancy been different than previous pregnancies?*

3. Next, I would like to know <u>what made you first think you might be pregnant</u> with this baby. Please describe any signs and symptoms that made you think this.
   a. Around when did you notice this? [*attempt to anchor in relation to last period*]
   b. Follow-up: Thinking back to that time, how did you feel about becoming pregnant? How do you feel now about being pregnant? How have your feelings changed over time?
      i. *Probe: How does your partner feel about you being pregnant? (happy, worried, unsure, concerned, stressed) How do you know he feels that way?*
   c. Follow-up: Had you been trying to become pregnant? Was the pregnancy a surprise?
   d. *Probe: What family planning method had you been using right before you became pregnant?*

4. Next, I would like to know <u>who you turned to when you first thought you were pregnant</u>. Please tell me who you talked to and shared that you thought you might be pregnant.
   a. Probe: for each person they mention – What are some of the reasons you choose that person to talk with? Are there any other reasons? Is it expected that you involve them? Did you turn to them because they are a source of emotional support or other kind of support? Do you turn to them because they are also a woman?
   b. Follow-up: What people did you <u>not</u> want to share that you thought you might be pregnant? What are some of the reasons that you did not want to turn to them? [Probe specifically about partner if did not share with them]. How soon after you thought you were pregnant did you share that information with your partner? What are the reasons you waited/didn't wait to tell him?
      i. *Probe: In what ways do you usually rely on that person?*

5. Next, I would like to know <u>how you confirmed that you were pregnant</u>. What were all the different things that you did to confirm that you were pregnant?
   a. *Probe: Ask about purchase of home pregnancy tests, visiting a health provider, testing at health center, and any other activities.*
      i. Probe: If used home pregnancy test- where did you buy it from? How much did it cost per test? How many did you buy? Had you used one before?
      ii. *Probe: If did not use a home pregnancy test – what are the reasons you did not use a home pregnancy test?*
      iii. *Probe: If visited a health provider – what did the provider do to confirm that you were pregnant?*
   b. Follow-up: How long after you first thought you were pregnant did you know for sure you were pregnant?

        i. *Probe: If there were delays – what are some of the reasons it took you that amount of time to confirm that you were pregnant?*

6. Once you knew that you were pregnant, I would like to know <u>who you turned to and shared that information</u>
    a. *Probe: for each person they mention – What are some of the reasons that made you choose that person to talk with? Are there any other reasons? Is it expected that you involve them? How so? Did you turn to them because they are a source of emotional support or other kind of support? Do you turn to them because they are also a woman?*
    b. Follow-up: What people did you <u>not</u> want to share that you were pregnant? What are some of the reasons that you did not want to turn to them? [Probe specifically about partner]
    c. Was there ever a time that you thought about terminating the pregnancy? *Probe: If so, who did you talk about this? What were the reasons you turned to them. Who did you not want to talk to about that? What were the reasons you did not want to talk to that person?*
        i. *Probe: Did you talk to your partner about this? Please tell me about the conversation – what did you say and what did he say?*
        ii. Follow-up: What were the reasons you decided not to terminate the pregnancy.

7. Next, I would like to learn more about <u>when you decided to first visit the health provider today for antenatal care</u> services. Please describe how you decided to first visit the health provider for antenatal care?
    a. *Probe: What were the main reasons you decided to visit the health provider for antenatal care? Were there any other reasons? [If so, what were they?]*
        i. *Follow-up: Did you decide to seek antenatal care services because you were experiencing complications? If so, what type of complications? How did the provider help?*
    b. *Probe: Who did you involve in helping you decide <u>when</u> to seek antenatal care? How did you involve them in that decision? What are the reasons you chose to involve that particular person?*
    c. *Probe: Which people did you <u>not</u> want to involve in this decision? For what reason did you not want to involve them?*
    d. Probe: Please describe any factors that may have led you to delay seeking antenatal care. Did being uncertain about whether you were pregnant cause any delays in seeking antenatal care? In what ways?
    e. *Probe: What type of provider did you see at this first visit? What happened during this first visit?*
    f. Follow-up: In total, how many antenatal care visits do you think you will make during this pregnancy? What makes you think you will have this number of visits? Are there reasons you would prefer having more or less visits? What reasons?

8. Next, I would like to know more about support, or resistance, from others for you to seek antenatal care. Please describe what kind of support or assistance others provided to enable you to visit the provider for this **first antenatal care visit.** By support I mean anything that people did to help, including financial or other ways (money, transportation or helping with responsibilities such as childcare or other work) or by providing emotional support or encouragement.
    a. *Probe: Who provided assistance? What kind of assistance or support did they provide?*
        i. *Probe: Ask specifically about:*
            1. *Emotional support (more detail; from whom?);*
            2. *Financial support (more detail; from whom?);*
            3. *Information (more detail; from whom?);*
            4. *Other support (e.g. childcare, transportation, other more detail; from whom?);*
        ii. *Probe: How important do you think that support was in helping you get the care she needed?*
        iii. *Ask specifically about partner.*
    b. *Probe: I'm interested in knowing more why you chose to involve that person. Is it expected that you involve them? How so? Did you turn to them because they are a source of emotional support or other kind of support? Do you turn to them because they are also a woman?*
    c. *Probe: If did not receive assistance or support – what are the reasons you did not receive any assistance or support to visit the provider? Ask specifically about partner if did not provide assistance.*
    d. Follow-up: Did anyone accompany you to your visit? [If someone went with her] What are the reasons you chose that person? How did they help you? [If no one went with her] What are the reasons that no one went with you? Would you have wanted that someone come with you? How could they have helped?
    e. Follow-up: What obstacles, if any, did you face in visiting the health provider for antenatal care? How did you overcome them?
        i. *Probe: What kind of support or assistance would you have wanted to have that you did not have? How would that support or assistance have helped you seek antenatal care services earlier, if at all?*
        ii. *Probe: Were there any individuals who made it harder for you to seek antenatal care? How did they make it harder? Please give me some examples.*
        iii. *Probe: Would you have wanted anyone to be more / less involved? Who? How so?*

9. Next, I would like to ask about whether you have sought antenatal care from any informal providers so far, such as a traditional healer, traditional birth attendant, or a community health worker. Please describe any other providers you visited so far for antenatal care – aside from those at the health center.

    Probe: What were the reasons that you sought care from those providers?

10. Next, I would like to ask about any concerns you have regarding testing you might receive at the antenatal care visits. Are there any concerns you have about being tested for HIV at the visits?
    a. *Probe: What kind of concerns do you have about HIV testing for you? [Example, partner rejection? Stigma?]*
    b. *Probe: Have you been tested for HIV recently? If so, have you shared that result with your partner? What reasons do you have to share/not share those results with your partner?*
    c. *Probe: Do you have concerns about transmitting HIV to your baby? What are your concerns? Are you interested in having your baby tested for HIV once the baby is born? What reasons do you have to want you baby to be tested or not? Who would be involved in making the decision about whether your baby was tested for HIV? {Probe for* mother, partner, sister, mother-in-law, friends, others] *Would the decision be her, yours or a joint decision?*
    d. *Probe: How comfortable do you feel talking about HIV with your partner? How comfortable do you feel talking with your partner about getting your child tested for HIV?*
    e. *Follow-up: With whom would you feel most comfortable discussing having your child tested for HIV? What are the reasons you would turn to that person? [Ask about different types of people such as partner, sister, mother, friend, health provider, health educator, another HIV-positive mother.] Are there people you would not want to turn to? What are the reasons you would not want to turn to them?*
    f. *Probe: Do you know other HIV-positive mothers who you could turn to for support if you needed? What are some examples of support that you would want from them if you needed?*

11. Next, I would like to hear what you think are the main benefits of antenatal care services?
    a. *Probe: In what ways do you think antenatal care visits help you during the pregnancy? What aspects of the visit are beneficial? What aspects of the visit are not beneficial?*
    b. Follow-up: When do you think a woman should have her first antenatal care visit?
    c. Follow-up: How frequently should a woman have antenatal care visits?
    d. Follow-up: Do you think antenatal care services are only for women who experience complications during pregnancy? What are some of the reasons you think that? Do you think women who have easier pregnancies without complications can also benefit from antenatal care services? How do you think they can benefit?
    e. Follow-up: It is recommended that women have at least 8 contacts with a health provider during their pregnancy. What do you think about this recommendation? How feasible would it be for you to visit a provider that number of times? *[Probe: Ask for more details about reasons it would or would not be feasible.]* What challenges would you have in having 8 contacts with a provider over the pregnancy? What would help make it more feasible?

12. Some women like to turn to other women in their community who have gone through similar experiences, such as pregnancy. Are there examples of women in your community who you have turned to during this or another pregnancy? How did you choose those women? In what ways have they influenced you? What did they help you with?
    a. Follow-up: If there were an opportunity for another mother to provide support to you during your pregnancy and post-partum, would you be interested in that? What kind of person would you want to have as support? What kind of support from her would help you?
    b. Follow-up: Have you heard of peer mothers who support mothers through pregnancy and after birth? What do you think about those types of programs? What would make you want to participate in that type of program? What would make you not want to participate?
    c. Follow-up: Who do you turn to for information about pregnancy? What are the main reasons you turn to them? Who do you turn to for information about the health of your baby? What are the main reasons? [Ask specifically about friends, sister, mother, mother-in-law, health providers, health educators, others.]

13. Next, I am interested to know at what point during the pregnancy would you want to be able to know that you are pregnant.
    a. *Probe: What are some reasons you might want to know earlier that you are pregnant? What are some reasons you might want to find out later that you are pregnant?*
    b. *Probe: If you found out earlier you were pregnant, how would that change your decisions about when to seek care? How would that change what services you decide to seek during the pregnancy?*
        i. *Probe: Would finding out earlier that you are pregnant lead you to seek antenatal care earlier? If not, what are other reasons that you might still delay seeking antenatal care?*
        ii. *Probe: Do you feel like women in your community have any reason to hide a pregnancy? What are the reasons? How do you feel about those reasons? At what point during a pregnancy would you feel comfortable sharing with others that you are pregnant?*
    c. Follow-up: Do you think it would be beneficial to seek antenatal care earlier in your pregnancy? How so? Do you think it would be beneficial to seek antenatal care more frequently during the pregnancy? How so?
    d. Follow-up: What do you think about home pregnancy tests? What has been your experience using them? If you have tests available at no-cost, how much sooner would you confirm your suspicions of pregnancy? Where would you like to be able to get a home pregnancy test? [probe for types of locations or venues] Would having access to low-cost home pregnancy tests affect when you seek health services? How would they affect that?

14. Please tell me about <u>your relationship with your partner</u>.

a. *Probe: How are things going, in your relationship?*
    i. *How is it the same now that you are pregnant?*
    ii. *How have things changed since you became pregnant [this time]?*
b. *Probe: Describe how your partner has been supportive during the pregnancy. How does he help you? Are there some ways you wish your partner were more supportive? Tell me about that.*

15. Next, I would like to hear more about how you and your partner make <u>decisions as a couple</u> about your health. Please describe how you make decisions when it comes to your health.
    a. Follow-up: When it comes to decisions about <u>your health</u>, in what ways do you feel you can make decisions on your own? In what ways do you feel you need to involve your partner? In what ways does he make the decisions on his own? [Ask about which health decisions]. others? How so?
        i. *Probe: Aside from your partner, please describe if there is anyone else who has a lot of influence on decisions about your health (positive or negative).*
    b. Follow-up: When it comes to decisions about your children's health, in what ways do you feel you like you can make those decisions on your own? In what ways do you make those decisions with your partner? In what ways does he make the decisions on his own? [Ask about which health decisions].
        i. *Probe: Aside from your partner, please describe if there is anyone else who has a lot of influence on decisions about your health (positive or negative).*
    c. Follow-up: Are there certain areas of your life where you have more say than your partner? What are those areas? Are there certain areas of your life where you do not need to involve your partner in decision? What are those areas? (*Probe for financial decisions, children, transportation, work, health.*

16. Next, I would like to learn more about <u>discussions with your partner</u> about different topics.
    a. Follow-up: How would you describe the communication between you and your partner in general? How about communication about pregnancy? Communication about your child's health? Communication about family planning?
    b. Follow-up: What topics related to family planning and pregnancy do you not feel comfortable discussing with your partner? What are some of the reasons you do not feel comfortable discussing those topics?

Is there anything else that you would like to tell me about your experience finding out you were pregnant and seeking antenatal care services? Is there anything that we did not talk about that you think would be important for me to know? Is there anything that other women should know when they first find out they are pregnant and are figuring out when to seek antenatal care?

Table S1. Characteristics of study participants

	**Single women (n=16) N (%)***	**Partnered women (n=15) N (%)**
Age		
18-19 years	8 (50%)	0 (0%)
20-24 years	5 (31%)	12 (80%)
25+ years	3 (19%)	3 (20%)
Education		
Some primary	3 (19%)	4 (27%)
Some secondary	11 (69%)	7 (47%)
Secondary or higher	2 (13%)	4 (27%)
Religion		
Anglican	3 (19%)	3 (20%)
Catholic	7 (44%)	3 (20%)
Muslim	3 (19%)	5 (33%)
Pentecostal/Born Again	3 (19%)	4 (27%)
Number of living children		
0	12 (75%)	8 (53%)
1	4 (25%)	5 (33%)
2	0 (0%)	0 (0%)
3+	0 (0%)	2 (13%)
Weeks pregnant		
≤13 weeks	1 (6%)	5 (33%)
14-26 weeks	7 (44%)	8 (53%)
27+ weeks	8 (50%)	2 (13%)
Previous abortion		
Yes	1 (6%)	2 (13%)
No	14 (94%)	14 (87%)
Considered abortion		
Yes	8 (50%)	5 (33%)
No	8 (50%)	10 (67%)

*Percent values are rounded to the nearest whole number and therefore some categories may not add up to exactly 100%.
